# Hypercalcémie maligne révélant une leucémie aiguë lymphoblastique : à propos d´un cas

**DOI:** 10.11604/pamj.2022.41.257.27209

**Published:** 2022-03-29

**Authors:** Karima Fakhri, Fatima Zahra Lahlimi, Illias Tazi

**Affiliations:** 1 Service d'Hématologie Clinique, Centre Hospitalier Universitaire Mohammed VI Marrakech, Faculté de Médecine et de Pharmacie, Université Cadi Ayyad, Marrakech, Maroc

**Keywords:** Hypercalcémie, leucémie, urgence, traitement, cas clinique, Hypercalcaemia, leukemia, urgency, treatment, case report

## Abstract

L´hypercalcémie maligne est une urgence métabolique, son association avec les tumeurs solides est fréquente alors qu´elle est rarement décrite dans les hémopathies malignes en dehors du myélome multiple et des leucémies/lymphomes T liées à HTLV-I. Nous rapportons le cas d´une patiente qui présente une leucémie aiguë lymphoblastique révélée par une hypercalcémie maligne et une fracture pathologique de l´humérus. Le bilan biologique a retrouvé une anémie normochrome normocytaire arégénérative, une hypercalcémie (163mg/l), une insuffisance rénale, créatinine (22mg/l) et un débit de filtration glomérulaire à 26 ml/min et un taux de parathormone (PTH) est bas (9,9pg/ml), la radiographie du membre supérieur droit a montré une fracture pathologique de l´humérus. Le myélogramme et l´immunophénotypage a confirmé le diagnostic de LAL-B. la patiente a bénéficié d´un traitement urgent de l´hypercalcémie et de la pathologie maligne sous-jacente. A travers cette observation nous voulons attirer l´attention sur la possibilité de ce tableau atypique au cours des leucémies aiguës et ses difficultés diagnostiques et thérapeutiques.

## Abstract (en)

Malignant hypercalcaemia is a metabolic emergency. Its association with solid tumors is common, whereas it has been rarely described in patients with malignant hemopathies other than multiple myeloma and T-cell leukemias/T-cell lymphomas associated with lymphotropic virus type I (HTLV-I). We here report the case of a female patient with acute lymphoblastic leukemia revealed by malignant hypercalcaemia and pathological fracture of the humerus. Laboratory tests showed aregenerative, normocytic-normochromi anemia, hypercalcaemia (163mg/l), renal failure, creatinine (22mg/l), glomerular filtration rate 26 ml/min and low parathyroid hormone (PTH) (9.9pg/ml). X-ray of the right upper limb showed pathological fracture of humerus. Myelogram and immunophenotyping confirmed the diagnosis of LAL-B. The patient received urgent treatment for hypercalcaemia and the underlying malignancy. This study focuses attention on this atypical condition occurring during acute leukemias and on its diagnostic and therapeutic issues.

## Introduction

La leucémie aiguë lymphoblastique (LAL) est une hémopathie maligne qui représente environ 20% des leucémies aiguës de l´adulte [1]. Son association à une hypercalcémie est exceptionnelle. Le tableau clinique associe habituellement un syndrome d´insuffisance médullaire et un syndrome tumoral d´installation brutale [2]. A travers cette observation, nous rapportons un cas d´une LAL révélée par une hypercalcémie maligne associée à une fracture pathologique de l´humérus.

## Patient et observation

**Information sur le patient:** une patiente de 39 ans, ayant un antécédent de goitre évoluant depuis 3 mois, présente depuis un mois des douleurs osseuses diffuses associées à des vomissements dans un contexte d´altération de l´état général sans autres signes associés.

**Résultats cliniques:** l´examen clinique retrouve une patiente avec un performance status à 4 avec de bonnes constantes hémodynamiques, une pâleur cuanéo-muqueuse, une impotence fonctionnelle du membre supérieur droit avec une douleur exquise à la palpation et une adénopathie inguinale droite mobile indolore de 1 cm sans signe inflammatoire en regard. Le reste de l'examen clinique est normal.

**Démarche diagnostique:** l´hémogramme objective une anémie à 5,6g/dl normochrome normocytaire arégénérative avec un taux de réticulocytes à 91500/mm^3^, une lymphopénie à 740/mm^3^, un taux de polynucléaires neutrophiles à 4433/mm^3^ ainsi qu´un taux normal de plaquettes à 391000/mm^3^, le frottis du sang périphérique n´a pas mis en évidence la présence de cellules suspectes. Devant les douleurs osseuses et l´anémie arégénérative, un ionogramme et un myélogramme est demandé, infiltré par 70% de blastes de taille moyenne, noyau à chromatine fine nucléolé et un cytoplasme basophile non granulaire à MPO négative concluant à une LAL de type 2. Un immunophénotypage réalisé retrouve des marqueurs lymphoïdes B positifs CD 19 (91%), CD 10 (68%). Les marqueurs myéloïdes et lymphoïdes T sont négatifs. Un premier ionogramme sanguin a montré une hypercalcémie (163mg/l) (90-105mg/l) confirmée par un deuxième dosage. Les dosages sanguins de phosphore (38 mg/L) et d´albumine (32 g/L) sont normaux. Le bilan rénal retrouve une insuffisance rénale avec une créatinine sérique à 22mg/l et un débit de filtration glomérulaire à 26 ml/min. Le taux de parathormone (PTH) est bas (9,9pg/ml) (Tableau 1). La radiographie thoracique est normale, sans élargissement du médiastin. La radiographie du membre supérieur droit a révélé une fracture pathologique de l´humérus. L´électrocardiographie retrouve une tachycardie sinusale sans signes électriques d´hypercalcémie. L´échographie cervicale objective un goitre suspect classé TIRADS 5, avec à la cytologie thyroïdienne des atypies de signification indéterminée (catégorie III). Le bilan préchimiothérapie, comportant une échographie cardiaque, les sérologies de VIH, hépatite C, hépatite B et la ponction lombaire, est normal.

**Tableau 1:** différents paramètres biochimiques de la patiente

Paramètres	Les valeurs	
	Avant le traitement	J3 après le traitement
Calcium	163 mg/l	102 mg/l
Phosphore	38 mg/l	36 mg/l
Albumine	32 g/l	35 g/l
Créatinine	22 mg/l	15 mg/l
DFG	26 ml/min	41 ml/min
Parathormone	9,9 pg/ml	-

**Intervention thérapeutique:** la prise en charge urgente de l´hypercalcémie a consisté en une hyperhydratation parentérale 3L/m^2^ par jour associée au biphosphonate (acide zolédronique 4mg à j1 et j3), avec surveillance de la diurèse permettent une normalisation progressive de la calcémie. La patiente a reçu une corticothérapie selon le protocole national à base de dexaméthasone 40mg/j pendant 4 jours avant l´initiation de la chimiothérapie.

**Suivi et résultats:** l´évolution a été marquée par l´aggravation du tableau clinique et l´installation d´un état de choc septique. La patiente a été mise sous bi-antibiothérapie en intraveineuse à base de ceftazidime (100mg/kg/j) associé à un aminoside (15mg/kg/j). Le bilan infectieux, incluant une hémoculture, un examen cytobactériologique des urines, coproculture, est stérile. Le décès est survenu deux jours plus tard.

**Consentement éclairé:** il a été obtenu de la patiente pour que nous puissions utiliser pour ce rapport de cas.

## Discussion

Les LAL représentent 20% des leucémies et 0,2% des néoplasies chez l´adulte [2]. L´hypercalcémie au cours des hémopathies malignes est un événement rare, en dehors du myélome multiple (13-30%), de leucémie T de l´adulte infecté par le virus HTLV-I (ATLL) (50-70%), du lymphome non hodgkinien (13%) et du lymphome hodgkinien (5,4%) [3]. Au cours des leucémies aiguës (LA), la fréquence de survenue de manifestations osseuses et/ou d´une hypercalcémie est plus élevée chez l´enfant et dans les LA lymphoblastiques. Les douleurs osseuses sont présentes dans 5% des cas lors du diagnostic et peuvent être révélatrices, comme le cas dans notre observation, ou apparaitre au cours de l´évolution dans 50% des cas [4].

L´hypercalcémie est rare, elle est présente dans moins de 1% des cas en concomitance avec une ostéolyse extensive. Lorsqu´elle est confirmée, il faut évaluer la sévérité afin d´adapter la thérapeutique. Les symptômes cliniques étant souvent tardifs : malaises, anorexie, nausées, vomissements, douleurs abdominales, constipation, syndrome polyuro-polydipsique, confusion voire coma [5]. Un dosage de la PHT intacte doit être réalisé afin d´éliminer les autres causes d´hypercalcémie telle que l´hyperparathyroïdie primitive. Le taux de PTH est souvent bas ou normal, c´est le cas dans notre observation. L´hypercalcémie est expliquée par deux mécanismes dans les hémopathies malignes : Une destruction osseuse localisée en rapport avec l´invasion tumorale osseuse ou médullaire et une stimulation de la résorption osseuse médiée par les protéines et les cytokines générées par les cellules néoplasiques ou par le microenvironnement [6]. Les principaux facteurs humoraux qui altèrent la régulation du calcium : la PTH related protein (PTHrP), les interleukines (IL -1, IL-6, IL11), le facteur de croissance alpha [TGF-a] et le facteur de nécrose tumorale alpha [TNF-a] [7]. La PTH related protein (PTHrP) est le facteur responsable du syndrome d'hypercalcémie humorale maligne entraînant une hypophosphorémie et une hypercalcémie par augmentation de la résorption osseuse, la réabsorption rénale du calcium et la perte rénale du phosphore [8]. Un dosage de PTHrP doit être réalisé devant une hypercalcémie non expliquée, un taux très élevé peut être prédictif d´une résistance au traitement par biphosphonates.

La prise en charge thérapeutique d´une hypercalcémie maligne repose sur deux principes : le traitement de la pathologie maligne sous-jacente et la diminution de la calcémie. La prise en charge urgente de l´hypercalcémie repose sur l´hydratation avec une solution saline normale (3L/m^2^ par jour) adaptée à la déshydratation avec une surveillance étroite de la diurèse. Les biphosphanates sont des puissants inhibiteurs de la résorption osseuse en bloquant l´activité des ostéoclastes, il faut les administrer dès que l´hypercalcémie est diagnostiquée. En cancérologie, les biphosphonates ont également montré un effet antalgique sur les douleurs osseuses. Le zoledronate est le biphosphonate actuellement le plus efficace, il est prescrit à la dose de 4 mg [9].

La calcitonine a un effet transitoire sur la calcémie par inhibition de la résorption osseuse et augmentation de l´excrétion urinaire de calcium. Elle peut être utilisée initialement avec les biphosphonates dans les cas de symptômes graves pour obtenir une réponse rapide [10]. La calcitonine s´administre à la dose de 4 à 8 unités internationales (UI)/kg/j, par voie sous cutanée, toutes les 6 à 12 h, elle a un effet rapide en quelques heures, mais elle est souvent mal tolérée. Les diurétiques de l´anse tel que le furosémide peuvent être administré selon les besoins et doivent être réservé lors de la surcharge résultant de l’hyperhydratation. Les corticoïdes, peuvent être utilisés en augmentant la production de calcitriol par les macrophages et diminuant l´absorption intestinale de calcium, le produit utilisé est l´hydrocortisone par voie intraveineuse à raison de 200 à 300 mg pendant 3 à 5 jours. Une épuration extra rénale doit être proposée en cas d´hypercalcémie sévère associée à une défaillance cardiaque ou rénale.

Le traitement de la pathologie maligne sous jacente repose principalement sur une chimiothérapie intensive comportant plusieurs phases. D´abord la phase d´induction (corticoïdes, vincristine, asparaginase, parfois anthracyclines), avec une prophylaxie cérébro-méningée (méthotrexate), puis la consolidation, l´intensification, et enfin la phase d´entretien.

## Conclusion

Notre observation soulève la question d'un lien possible entre l´hypercalcémie et la leucémie aigue lymphoblastique. Cette association a été rarement décrite dans la littérature. Le diagnostic de l´hypercalcémie lié aux hémopathies malignes n´est retenu qu´après élimination d´une hyperparathyroïdie associée. La prise en charge nécessite un traitement en urgence de l´hypercalcémie et de la pathologie maligne sous jacente.
